# Trans-ethnic genome-wide association study of severe COVID-19

**DOI:** 10.1038/s42003-021-02549-5

**Published:** 2021-08-31

**Authors:** Peng Wu, Lin Ding, Xiaodong Li, Siyang Liu, Fanjun Cheng, Qing He, Mingzhong Xiao, Ping Wu, Hongyan Hou, Minghui Jiang, Pinpin Long, Hao Wang, Linlin Liu, Minghan Qu, Xian Shi, Qin Jiang, Tingting Mo, Wencheng Ding, Yu Fu, Shi Han, Xixiang Huo, Yingchun Zeng, Yana Zhou, Qing Zhang, Jia Ke, Xi Xu, Wei Ni, Zuoyu Shao, Jingzhi Wang, Panhong Liu, Zilong Li, Yan Jin, Fang Zheng, Fang Wang, Lei Liu, Wending Li, Kang Liu, Rong Peng, Xuedan Xu, Yuhui Lin, Hui Gao, Limei Shi, Ziyue Geng, Xuanwen Mu, Yu Yan, Kai Wang, Degang Wu, Xingjie Hao, Shanshan Cheng, Gaokun Qiu, Huan Guo, Kezhen Li, Gang Chen, Ziyong Sun, Xihong Lin, Xin Jin, Feng Wang, Chaoyang Sun, Chaolong Wang

**Affiliations:** 1 Department of Obstetrics and Gynecology, Tongji Hospital, Tongji Medical College, Huazhong University of Science and Technology, Wuhan, China; 2 National Medical Center for Major Public Health Events, Huazhong University of Science and Technology, Wuhan, China; 3 Department of Epidemiology and Biostatistics, School of Public Health, Tongji Medical College, Huazhong University of Science and Technology, Wuhan, China; 4 Ministry of Education Key Laboratory of Environment and Health, State Key Laboratory of Environmental Health (Incubating), School of Public Health, Tongji Medical College, Huazhong University of Science and Technology, Wuhan, China; 5 Hepatic Disease Institute, Hubei Key Laboratory of Theoretical and Applied Research of Liver and Kidney in Traditional Chinese Medicine, Hubei Provincial Hospital of Traditional Chinese Medicine, Wuhan, China; 6 Hubei Provincial Academy of Traditional Chinese Medicine, Wuhan, China; 7 School of Public Health (Shenzhen), Sun Yat-sen University, Shenzhen, Guangdong China; 8 Department of Hematology, Union Hospital, Tongji Medical College, Huazhong University of Science and Technology, Wuhan, China; 9 The Third People’s Hospital of Shenzhen, National Clinical Research Center for Infectious Disease, The Second Affiliated Hospital of Southern University of Science and Technology, Shenzhen, China; 10 Department of Laboratory Medicine, Tongji Hospital, Tongji Medical College, Huazhong University of Science and Technology, Wuhan, China; 11 Department of Occupational and Environmental Health, School of Public Health, Tongji Medical College, Huazhong University of Science and Technology, Wuhan, China; 12 Hubei Provincial Center for Disease Control and Prevention, Wuhan, China; 13 College of Life Sciences, University of Chinese Academy of Sciences, Beijing, China; 14 Department of Emergency, Union Hospital, Tongji Medical College, Huazhong University of Science and Technology, Wuhan, China; 15 Department of Pediatrics, Union Hospital, Tongji Medical College, Huazhong University of Science and Technology, Wuhan, China; 16 Department of Biostatistics, Harvard T. H. Chan School of Public Health, Boston, MA USA; 17 Department of Statistics, Harvard University, Cambridge, MA USA; 18 Broad Institute of MIT and Harvard, Cambridge, MA USA; 19 School of Medicine, South China University of Technology, Guangzhou, China

**Keywords:** Viral infection, Genome-wide association studies

## Abstract

COVID-19 has caused numerous infections with diverse clinical symptoms. To identify human genetic variants contributing to the clinical development of COVID-19, we genotyped 1457 (598/859 with severe/mild symptoms) and sequenced 1141 (severe/mild: 474/667) patients of Chinese ancestry. We further incorporated 1401 genotyped and 948 sequenced ancestry-matched population controls, and tested genome-wide association on 1072 severe cases versus 3875 mild or population controls, followed by trans-ethnic meta-analysis with summary statistics of 3199 hospitalized cases and 897,488 population controls from the COVID-19 Host Genetics Initiative. We identified three significant signals outside the well-established 3p21.31 locus: an intronic variant in *FOXP4-AS1* (rs1853837, odds ratio OR = 1.28, *P* = 2.51 × 10^−10^, allele frequencies in Chinese/European AF = 0.345/0.105), a frameshift insertion in *ABO* (rs8176719, OR = 1.19, *P* = 8.98 × 10^−9^, AF = 0.422/0.395) and a Chinese-specific intronic variant in *MEF2B* (rs74490654, OR = 8.73, *P* = 1.22 × 10^−8^, AF = 0.004/0). These findings highlight an important role of the adaptive immunity and the ABO blood-group system in protection from developing severe COVID-19.

## Abstract (web summary)

Chaolong Wang and colleagues report a large genome-wide association study for COVID-19 severity in Chinese individuals. By meta-analysis with European data, they identify 3 loci associated with severe disease that suggest key roles for the adaptive immune system and the ABO blood group system in development of severe COVID-19.

## Introduction

The coronavirus disease 2019 (COVID-19) is an ongoing pandemic caused by the severe acute respiratory syndrome coronavirus 2 (SARS-CoV-2). Despite a huge number of cases have been diagnosed, both modeling studies and seroprevalence studies estimate the actual number of infections to be much larger, suggesting the majority of infected individuals might have mild or no symptoms^1–4^. COVID-19 patients display a wide spectrum of clinical symptoms. Up to 5% of the confirmed cases would develop severe pneumonia with acute respiratory distress syndrome (ARDS)^5,6^ and millions of deaths have been attributed to COVID-19^7^. While older age, male sex, and comorbidities, such as hypertension, diabetes, obesity, and cardiovascular diseases, were found to associate with severe COVID-19^6,8,9^, many patients with no major risk factors were reported to develop severe symptoms^10^. Host genetic variation might contribute to the diverse clinical presentations of infectious diseases, potentially through the regulation of the immune system. Classic examples include the association between *CCR5* gene and the human immunodeficiency virus (HIV) infection^11^, *ABO* and malaria^12^ and SARS^13^, and *HLA-C* and chronic hepatitis B virus infection^14^.

The first genome-wide association study (GWAS) of COVID-19 has reported two severity-associated loci in Italians and Spanish: the 3p21.31 locus containing several immune genes and the *ABO* (9q34.2) locus determining ABO blood groups^15^. The 3p21.31 locus has been replicated by several follow-up studies, including the COVID-19 Host Genetics Initiative (HGI)^16^ and a recent GWAS comparing COVID-19 patients from intensive care units (ICU) across the UK and ancestry-matched population controls^17^, which also reported three additional loci at 12q24.13, 19p13.3, and 21q22.1. Furthermore, whole-genome sequencing studies (WGS) have identified several rare putative loss-of-function (LOF) variants, which could impair type I and II interferon (IFN) immunity, in association with severe COVID-19^18,19^.

Identification of host genetic variants associated with severe COVID-19 can help understand how our immune system interacts with SARS-CoV-2 and thus guide the development of effective prevention and therapeutic strategies, including prioritizing high-risk populations for vaccination in shortage of vaccine supply. Current genetic studies of COVID-19, like many other human genetic studies, are mainly based on European populations, which might lead to potential bias in translating findings to non-Europeans^20^. A striking example is the 3p21.31 locus for COVID-19. The risk haplotype at this locus was found to inherit from Neanderthals, reaching a high frequency of 30% in South Asians and 8% in Europeans, but almost absent in Africans and East Asians^15,21^. Thus, risk stratification based on this locus is not applicable to Africans and East Asians. A recent study of the host genetic contribution to COVID-19 severity in the Chinese population did not identify genome-wide significant association signal due to a small sample size of 332 patients^22^.

In this study, we bridge the gap by collecting and analyzing GWAS and WGS data of 1072 severe COVID-19 cases and 3875 controls (including 1526 patients with mild symptoms and 2349 population controls), all of the Chinese ancestry, and meta-analyzing with summary statistics from the HGI analysis (B2_release3) of 3199 hospitalized cases and 897,488 population controls of primarily European ancestry. We group population controls with mild patients because the vast majority of population controls would likely have COVID-19 with mild or no symptoms if they were exposed to the virus^1–4^. Our analyses lead to three significant loci predisposing risk to severe COVID-19, including an intronic variant in *FOXP4-AS1*, a frameshift insertion in *ABO*, and a Chinese-specific rare intronic variant in *MEF2B*.

## Results

We successfully genotyped 1626 COVID-19 patients from Tongji Hospital and Hubei Hospital of Traditional Chinese Medicine (TCM) in Wuhan using the Illumina Global Screening Array (GSA). After quality controls, we merged the COVID-19 dataset with 1459 population controls from the Coke Oven Worker (COW) cohort in Wuhan, who were genotyped using the same array, resulting in 369,072 autosomal and 8942 X chromosomal SNPs with minor allele frequency (MAF) > 0.005 in both datasets. The merged data were imputed to an East Asian WGS reference panel combining East Asians from the 1000 Genomes Project (1KGP)^23^ and our in-house WGS data of Chinese, achieving good imputation quality for common variants with MAF > 0.01 (“Methods” and Supplementary Fig. 1). We kept 6,019,210 autosomal and 132,535 X chromosomal variants with imputation *R*^2^ > 0.8 and MAF > 0.01 for downstream analyses.

After excluding second-degree and above relatedness and contaminated samples, we performed a GWAS of 598 severe cases versus 2260 controls (including 859 mild patients and 1401 population controls), correcting for the first two principal components (PCs) of population structure. The demographic characteristics of the samples were presented in Table 1. We detected a significant association signal located on 1p36.31, an intronic region of *CHD5* (rs34308690, OR = 1.50, *P* = 4.52 × 10^−8^, AF = 0.386, 0.389, and 0.144 in Chinese GWAS, Chinese WGS, and 1KGP Europeans, respectively) (Supplementary Fig. 2). We then combined our results with GWAS summary statistics of 3199 hospitalized COVID-19 patients versus 897,488 population controls from the HGI study (B2_release3). Meta-analysis of our GWAS and the HGI results did not replicate the signal in *CHD5*, but led to three other significant loci (*P* < 5 × 10^−8^), including the previously reported 3p21.31 locus and the *ABO* locus^15^, and a novel locus at 6p21.1 within the *FOXP4*-*AS1* gene (Supplementary Fig. 2). We noted that the signal of the 3p21.31 locus was solely from the HGI analysis because the risk variant at this locus was absent in our GWAS dataset^15,21^.

**Table 1:** Demographic characteristics of Chinese GWAS and WGS datasets.

	Chinese GWAS			Chinese WGS		
	*n*	Male (%)	Age (mean ± SD)	*n*	Male (%)	Age (mean ± SD)
Severe COVID-19	598	49.7	63.6 ± 12.3	474	54.9	61.5 ± 13.8
Mild COVID-19	859	45.6	55.8 ± 14.3	667	46.0	49.4 ± 16.2
Population control	1401	86.2	41.7 ± 8.1	948	47.4	29.0 ± 5.4

**Fig. 1:**
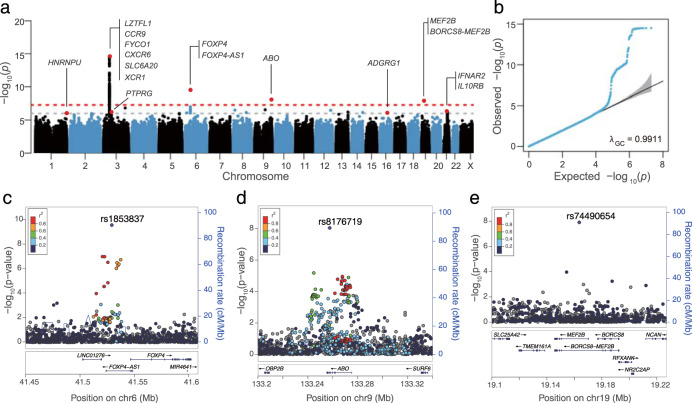
Trans-ethnic meta-analysis results for severe COVID-19. **a** Manhattan plot of meta-analysis *P* values. The red dash line indicates the genome-wide significance level at *P* = 5 × 10^−8^ and the gray dash line indicates the suggestive significance level at *P* = 10^−6^. **b** QQ plot, in which the gray region represents 95% confidence interval under the null hypothesis of no association. **c**–**e** Regional plots of three significant loci at 6p21.1, 9q34.2, and 19q13.11. The lead variant within each locus is indicated by the purple diamond while neighboring variants were colored based on LD to the lead variant in our Chinese WGS samples. Gray color indicates LD information is not available.

We then pooled together WGS data of 474 severe and 667 mild COVID-19 patients from Wuhan Tongji Hospital, Wuhan Union Hospital, and the Third People’s Hospital of Shenzhen, and 948 ancestry-matched population controls from BGI-Shenzhen, all sequenced at BGI (Shenzhen). These WGS samples have no overlap with our GWAS samples (Table 1 and “Methods”). Samples from different sources were sequenced in batches, in which 467 COVID-19 patients from Union Hospital were sequenced using cell-free DNA (cfDNA) to ~17.8× , while the other samples were sequenced to >33× following standard WGS protocols. To minimize batch effects, we performed linkage disequilibrium (LD) based joint calling and stringent variant quality controls, followed by association tests correcting for the first two PCs and an indicator variable for the cfDNA batch. Summary statistics from these WGS samples were meta-analyzed with those from the previous two GWAS datasets (Fig. 1a–e). We observed no genomic inflation in all association analyses (genomic inflation factor *λ*_GC_ < 1.011, Supplementary Fig. 2).

Lead variants in both the 6p21.1 locus and the *ABO* locus have a consistent direction of effect in the WGS samples, and no heterogeneity in effect size was detected across cohorts (Table 2). The top association signal at the 6p21.1 locus is an intronic SNP (rs1853837, alleles: C/A) of the *FOXP4-AS1* gene, which has *P* = 2.51 × 10^−10^ and OR = 1.28 (95% confidence interval [CI]: 1.19–1.39) after meta-analysis. The risk allele A is much more common in Chinese than in Europeans (allele frequency AF = 0.353 in our WGS Chinese and 0.105 in 1KGP Europeans). The top association signal at the *ABO* locus is a frameshift insertion in the *ABO* gene (rs8176719, alleles: T/TC, *P* = 8.98 × 10^−9^, OR = 1.19 [1.12–1.26]), which is the only variant passing the genome-wide significance level of *P* < 5 × 10^−8^ and is located about 6 kb away from the previously reported lead SNP rs657152 (LD in Chinese, *r*^2^ = 0.95)^15^. The rs8176719 insertion is common in both Europeans and Chinese (AF > 0.39). Notably, this variant is one of the three major variants determining the haplotypes of the ABO blood group^15,24,25^. Carriers of the T/T homozygote belong to blood group O, while carriers of the risk allele TC belong to the non-O group unless they also carry an extremely rare allele T at SNP rs41302905 (absence in our Chinese samples). Thus, consistent with previous studies^15^, our result suggests the blood group O is protective for COVID-19 severity. When further adjusting for age and sex (Supplementary Table 1 and Supplementary Fig. 3), the *FOXP4-AS1* locus remained significant (*P* = 4.20 × 10^−10^), but the signal at the *ABO* locus diminished (*P* = 5.88 × 10^−7^), likely due to loss of power given that our population controls were younger and mostly males (Table 1).

**Table 2:** Significant loci associated with COVID-19 severity.

Locus	Dataset	Sample size	Lead variant^a^	AF^b^	OR (95% CI)^c^	*P*	Heterogeneity
3p21.31	Chinese (GWAS)	598/2260	rs35044562	–	–	–	
*LZTFL1*	HGI (B2_release3)	3199/897,488	chr3:45867532	0.080	1.60 (1.42–1.79)	3.11 × 10^−15^	
	Chinese (WGS)	474/1615	A/G, Intronic	–	–	–	
6p21.1	Chinese (GWAS)	598/2260	rs1853837	0.345	1.30 (1.13–1.50)	3.24 × 10^−4^	
*FOXP4-AS1*	HGI (B2_release3)	3199/897,488	chr6:41529297	0.105	1.28 (1.15–1.42)	5.24 × 10^−6^	
*FOXP4*	Chinese (WGS)	474/1615	C/A	0.353	1.27 (1.07–1.51)	7.06 × 10^−3^	*I*^2^ = 0.00%
	Meta-analysis	4271/901,363	Intronic		1.28 (1.19–1.39)	2.51 × 10^−10^	*P*_het_ = 0.97
9q34.2	Chinese (GWAS)	598/2260	rs8176719	0.422	1.28 (1.12–1.46)	3.19 × 10^−4^	
*ABO*	HGI (B2_release3)	3199/897,488	chr9:133257521	0.395	1.17 (1.09–1.26)	1.27 × 10^−5^	
	Chinese (WGS)	474/1615	T/TC	0.433	1.17 (0.98–1.38)	8.03 × 10^−2^	*I*^2^ = 0.00%
	Meta-analysis	4271/901,363	Exonic (frameshift)		1.19 (1.12–1.26)	8.98 × 10^−9^	*P*_het_ = 0.51
19q13.11	Chinese (GWAS)	598/2260	rs74490654	–	–	–	
*MEF2B*	HGI (B2_release3)	3199/897,488	chr19:19163581	–	–	–	
	Chinese (WGS)	474/1615	C/G, Intronic	0.004	8.73 (4.14–18.41)	1.22 × 10^−8^	

Notes: Sample size is presented as the number of cases/number of controls.

^a^Variant with the smallest *P*-value within each locus: rs number, GRCh38 genomic position, reference/alternative alleles, and annotation of the variant.

^b^AF: frequency of the alternative allele: the first row is based on controls from the Chinese GWAS samples, the second row is based on the 1KGP European samples, and the third row is based on controls from Chinese WGS samples.

^c^Odds ratio (OR) and 95% confidence interval (CI) of the alternative allele. Meta-analysis is based on the Han-Eskin random-effect method^63^. Gene expression patterns for *FOXP4-AS1*, *FOXP4*, *ABO*, and *MEF2B* from GTEx^28^ are shown in Fig. 3a–d.

**Fig. 2:**
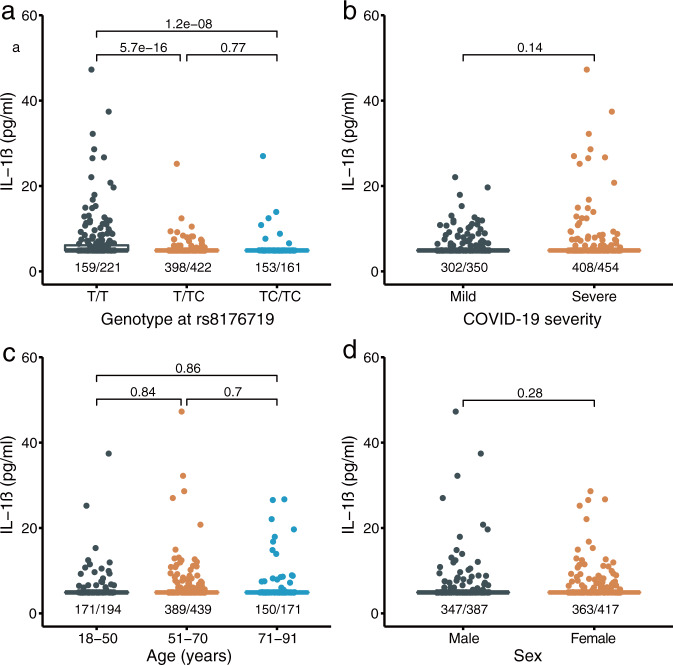
Comparison of serum level of IL-1β in different groups. Serum level of IL-1β between groups defined by the genotype at rs8176719 (**a**), COVID-19 severity (**b**), age (**c**), and sex (**d**). *P* values between groups, which are indicated above the horizontal line, were derived from Wilcoxon rank-sum tests. The number of samples below the limit of detection (5 pg/ml) and the total number of samples in each group are indicated below the box plot, separated by “/”. Source data are provided in Supplementary Data 1.

To explore the potential mechanism of these two common variant association signals, we examined the association between SNP genotypes and nine inflammatory biomarkers in a subset of COVID-19 patients with detailed clinical data during their hospitalization. These serum biomarkers included interleukin 1 beta (IL-1β, sample size *n* = 804), interleukin 2 receptor (IL-2R, *n* = 802), interleukin 6 (IL-6, *n* = 846), interleukin 8 (IL-8, *n* = 790), interleukin 10 (IL-10, *n* = 799), tumor necrosis factor-alpha (TNF-α, *n* = 785), complements C3 (*n* = 273) and C4 (*n* = 272), and C-reactive protein (CRP, *n* = 768). Interestingly, we observed a significant association between genotypes of rs8176719 at the *ABO* locus and the serum level of IL-1β (Spearman’s correlation *r*_s_ = −0.274, *P* = 2.61 × 10^−15^, Fig. 2a), despite no association between IL-1β and COVID-19 severity in our samples (Table 3 and Fig. 2b). The association between rs8176719 and IL-1β remains significant after dichotomizing IL-1β as normal (≤12 pg/ml) and abnormal (> 12 pg/ml) and controlling for COVID-19 severity, age, and sex (OR = 0.22 [0.10–0.50], *P* = 2.28 × 10^−4^, Wald test based on logistic regression, Fig. 2b–d). We found no association between rs1853837 at *FOXP4-AS* and inflammatory biomarkers.

**Table 3:** Serum levels of inflammatory biomarkers in Chinese COVID-19 patients.

Clinical variable	Sample size	Range^a^ (min, max)	No. of samples below LOD	Association with covariates and genotypes [Spearman correlation (*P* value)]^b^				
				Severity	Age	Sex	rs1853837	rs8176719
IL-1β	804	(5.00, 47.30)	710	−0.052 (0.140)	−0.005 (0.890)	0.038 (0.276)	−0.046 (0.194)	−0.274 (2.61 × 10^−15^)
IL-2R	802	(54.00, 5454.00)	0	0.371 (1.49 × 10^−27^)	0.293 (2.52 × 10^−17^)	−0.182 (2.26 × 10^−7^)	0.059 (0.092)	0.068 (0.056)
IL-6	846	(1.50, 5000.00)	222	0.435 (1.89 × 10^−40^)	0.387 (1.13 × 10^−31^)	−0.169 (7.52 × 10^−7^)	0.026 (0.448)	0.029 (0.399)
IL-8	790	(5.00, 462.00)	175	0.136 (1.20 × 10^−4^)	0.123 (5.34 × 10^−4^)	−0.124 (4.59 × 10^−4^)	0.017 (0.634)	−0.018 (0.615)
IL-10	799	(5.00, 436.00)	579	0.186 (1.14 × 10^−7^)	0.045 (0.199)	−0.105 (0.003)	0.003 (0.930)	−0.002 (0.953)
TNF-α	785	(4.00, 194.00)	105	0.154 (1.38 × 10^−5^)	0.167 (2.46 × 10^−6^)	−0.176 (6.96 × 10^−7^)	0.067 (0.061)	0.011 (0.765)
C3	273	(0.12, 1.45)	0	−0.080 (0.188)	−0.171 (0.005)	0.037 (0.545)	0.169 (0.005)	0.002 (0.970)
C4	272	(0.02, 1.32)	0	0.107 (0.077)	−0.078 (0.197)	−0.171 (0.005)	0.047 (0.440)	−0.046 (0.450)
CRP	768	(0.10, 320.00)	0	0.506 (4.44 × 10^−51^)	0.225 (2.79 × 10^−10^)	−0.186 (2.11 × 10^−7^)	0.109 (0.002)	0.043 (0.230)

^a^Minimum value of the range indicates the lower limit of detection (LOD) when there are samples below LOD.

^b^Severity is coded as 0 for mild and 1 for severe patients. Sex is coded as 1 for male and 2 for female. Genotypes are coded as 0, 1, and 2 for the copies of the alternative allele.

In addition, we identified a rare intronic SNP within the *MEF2B* gene reaching genome-wide significance (rs74490654 at 19q13.11, alleles: C/G, *P* = 1.22 × 10^−8^). This signal is contributed by our Chinese WGS data because the alternative allele is extremely rare with AF = 0.4% in 1KGP East Asians and 0 in other continental groups^23^. The AF in our WGS controls (including mild patients) is 0.4%, the same as the 1KGP East Asians, but increases to 2.2% in our cases with severe COVID-19. Among 35 carriers of the alternative allele (all in heterozygote), there are 21 from 474 severe cases, 4 from 667 controls with mild COVID-19, and 10 from 948 population controls, translated into a large effect size of OR = 8.73 (95% CI: 4.14–18.41). Variant rs74490654 locates only 7 bp upstream of an ENCODE candidate *cis*-regulatory element (cCRE) E1945041, which is annotated with a distal enhancer-like signature in the B-cell lymphocyte lineage OCILY7^26^. Hi-C data further suggest the cCRE E1945041 and the promoter of *MEF2B* locate in the same topologically associating domain (TAD)^27^, indicating rs74490654 is likely to disrupt the transcriptional activities of *MEF2B*.

Finally, there are four suggestively significant loci (*P* < 10^−6^) reported by at least two datasets (Supplementary Table 2): 21q22.11 (rs1051393 in *IFNAR2*, OR = 1.17 [1.10–1.25], *P* = 4.33 × 10^−7^), 3p14.2 (rs672699 in *PTPRG*, OR = 1.18 [1.04–1.34], *P* = 5.58 × 10^−7^), 16q21 (rs7499679 in *ADGRG1*, OR = 0.85 [0.79–0.90], *P* = 8.09 × 10^−7^), and 1q44 (rs12130553 in *HNRNPU*, OR = 1.19 [1.11–1.27], *P* = 9.17 × 10^−7^). All four loci have a consistent direction of effects across datasets, but locus 3p14.2 (rs672699) has significant variation in the effect sizes (*I*^2^ = 67.21%, *P*_het_ = 0.05; Supplementary Table 2).

## Discussion

In this study, we tested host genetic association with COVID-19 severity based on the largest COVID-19 GWAS and WGS datasets of Chinese ancestry to date, including 1072 severe COVID-19 patients, 1526 mild patients, and 2349 population controls. We detected a Chinese-specific rare variant (rs74490654 in *MEF2B* 19q13.11) at genome-wide significance in the WGS samples of 474 cases and 1615 controls. This signal, however, was not replicated in the GWAS samples due to the low imputation quality of rare variants. Given the limited power to detect rare variant association with our small WGS sample size, further sequencing-based replication in large samples will be required to confirm the association signal at *MEF2B*. Two additional loci (*FOXP4-AS1* at 6p21.1 and *ABO* at 9q34.2) were identified by trans-ethnic meta-analysis with summary statistics from the HGI (B2_release3) analysis of 3199 hospitalized patients and 897,488 population controls, most of which were European samples, highlighting that COVID-19, like many other complex diseases, requires a large sample size to reliably detect moderate effects of common variants.

In the HGI analysis, cases were hospitalized COVID-19 patients and controls were population controls with no information on COVID-19. Given that most hospitalized patients in western countries have severe symptoms and that most SARS-CoV-2 infections result in mild or no symptoms, the HGI data likely enrich for genetic associations with COVID-19 severity, despite different case definitions from our samples. The only significant locus in the HGI B2_release3 analysis was the 3p21.31 locus, which was first identified in Italians and Spanish^15^. Because the risk haplotype of this locus was almost absent in East Asians, our data provide no additional evidence.

The lead SNP of locus 6p21.1, rs1853837, is in the intron of the lncRNA forkhead box P4 antisense RNA 1 (*FOXP4-AS1*). The risk allele (A) at rs1853837 is an eQTL in positive association with the expression of *FOXP4-AS1* in lung^28^, and has been reported to associate with an increased risk for non-small cell lung cancer by GWAS^29^. The GeneHancer database indicates rs1853837 is resided in an enhancer targeting *FOXP4-AS1*, forkhead box P4 (*FOXP4*), and natural cytotoxicity triggering receptor 2 (*NCR2*)^30^.

During the revision of this paper, an updated HGI analysis with a much larger sample size (B2_release5, involving 13,641 cases and 2,070,709 controls) has identified *FOXP4-AS1* as a significant locus associated with hospitalized COVID-19 (rs1886814, OR = 1.26, *P* = 1.11 × 10^−9^, LD *r*^2^ = 0.64 with rs1853837)^31^, supporting the validity of our result. *FOXP4* is a transcription factor expressed in thymocytes and peripheral CD4+ and CD8+ T cells, and knockout of *FOXP4* can impair memory recall of T-cell cytokines in response to viral infections^32^. For COVID-19, SARS-CoV-2-specific T cells have been detected in many uninfected healthy individuals, likely due to an exposure history to common cold coronaviruses^33,34^. Such cross-reactive T-cell immunity from other coronavirus has been speculated to affect COVID-19 severity. Furthermore, FOXP4 plays a key role in regulating lung secretory epithelial cell fate and regeneration and thus can affect the production of mucus to protect the lung against pathogens and pollution^35^.

The association between blood group O with COVID-19 has been reported by both genetic and non-genetic studies^15,25,36,37^. However, as discussed by Ellinghaus et al.^15^, their association signal at the *ABO* gene (9q34.2) might be subject to population stratification because of the inclusion of blood donors as controls, which might enrich for blood group O. Skepticism of this association was elevated when the association was not replicated in the HGI release 3 analysis with large sample size. Our result, in contrast, confirmed the association at a frameshift insertion rs8176719 of the *ABO* gene, which showed no heterogeneity across populations (OR = 1.28 [1.12–1.46] in Chinese GWAS, 1.17 [1.09–1.26] in HGI B2_release3, and 1.17 [0.98–1.38] in Chinese WGS; *I*^2^ = 0.00%, *P*_het_ = 0.51). rs8176719 is the major variant determining blood group O and has been reported to associate with susceptibility to malaria^12^. The ABO blood groups have also been implicated in the association with susceptibility to SARS^13^ and several immune diseases, such as allergy, amyotrophic lateral sclerosis, and asthma^38–40^. We observed that carriers of T/T homozygote at rs8176719 (i.e., individuals of blood group O) tend to have an elevated serum level of IL-1β among COVID-19 patients (Fig. 2b). Nevertheless, unlike other inflammatory cytokines, such as IL-2R, IL-6, IL-8, IL-10, TNF-α, and CRP, which were elevated in severe patients due to strong immune response, we observed no association between IL-1β and COVID-19 severity (Table 3). Further investigations are needed to understand how the *ABO* gene affects susceptibility to severe COVID-19.

**Fig. 3:**
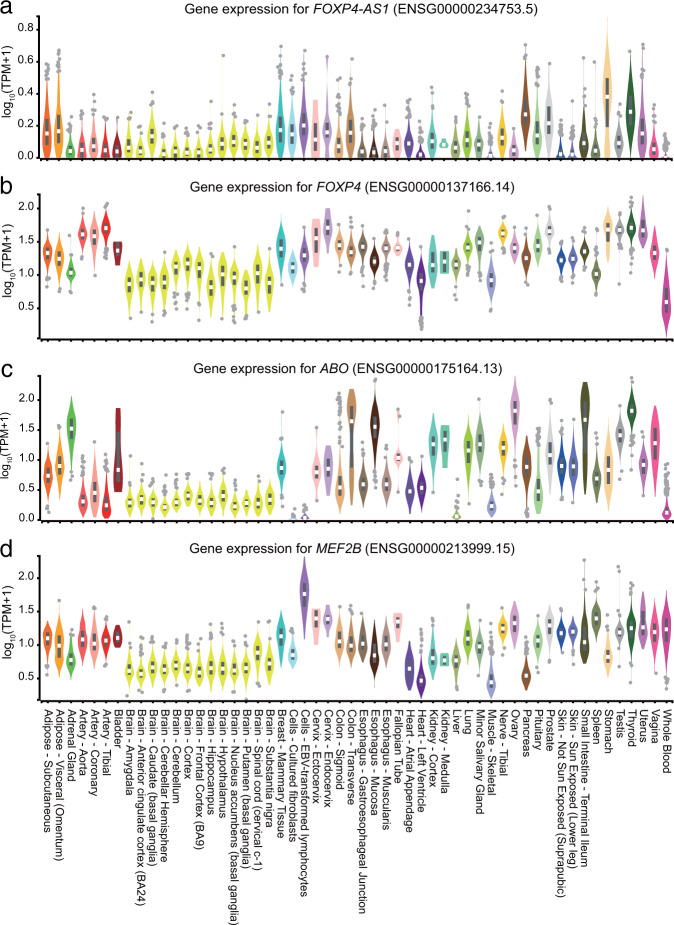
RNA expression in multiple tissues for genes within significant loci. **a***FOXP4-AS1*. **b***FOXP4*. **c***ABO*. **d***MEF2B*. TPM, transcripts per million. This figure was generated by the GTEx portal (https://www.gtexportal.org).

The top SNP rs74490654 was a rare variant located in the intron of myocyte enhancer factor-2B (MEF2B), one of the four MEF2 transcription factors involved in the regulation of muscle, neural crest, endothelial cell, and lymphocyte development^41^. Both epigenetic and Hi-C annotations suggest rs74490654 is likely to transcriptionally regulate the expression of *MEF2B* in lymphocytes. MEF2B could bind to its target DNA sites with a degenerate motif and act as a specific transcriptional regulator^42^. Importantly, MEF2B is overexpressed in lymphocytes (Fig. 3d)^28^ and plays critical roles in anti-virus immune which would be associated with COVID-19 development and severity. First, MEF2B is critical for the formation of germinal centers and promoting early B-cell development^43,44^, while B cells are essential in the defense of virus infection by producing protective antibodies. Second, MEF2 is necessary during peripheral T-cell activation by activating IL-2 and other cytokines^45,46^. Moreover, Clark et al. revealed that MEF2 regulates susceptibility to infection and is associated with tolerance of pathology by regulating metabolism^47^. Taking together, MEF2 plays essential roles in B-cells development, T-cells activation, and in immune-metabolic switch, which could at least partially explain the association between rs74490654 and COVID-19 severity.

Several candidate genes within the four suggestive loci have been reported to associate with immune-related traits. *IFNAR2* and *IL10RB* at 21q22.11 were associated with the susceptibility to hepatitis B virus infection^48^, Crohn’s disease^49^, type 2 diabetes^50^, and immunodeficiency^51^. In particular, *IFNAR2* encoded interferon alpha- and beta-receptor subunit 2, which is essential for antiviral immunity^52^. Both common and rare variants in *IFNAR2* have been implied in the susceptibility to severe COVID-19^17,19^. *PTPRG* at 3p14.2 was associated with pneumococcal bacteremia^53^. While *HNRNPU* at 1q44 is famous for association with neurodevelopmental delays and epilepsy^54,55^, it also plays a role in restricting HIV activity by blocking the cytoplasmic accumulation of viral mRNA transcripts^56^.

Vigorous multi-faceted public health interventions have led to rapid control of the COVID-19 epidemic in China^3,57^. Therefore, it is difficult for us to recruit more patients to increase the sample size and statistical power. We augmented our samples with population controls from two existing cohorts, who were primarily males (for the COW cohort) and under age 50 (for both). To avoid loss of power, we did not adjust for age and sex in our main analyses because age and sex were systematically different between cases and population controls due to data collection rather than biological effects. Given that age and sex are not associated with autosomal genotypes, we do not expect spurious genetic association signals due to confounding effects of unadjusted age and sex. We identified common variants in *ABO* and *FOXP4-AS1* and a rare variant in *MEF2B* to be significantly associated with COVID-19 severity, likely through regulation of the adaptive immunity. Unlike the 3p21.31 locus, of which the risk haplotype is specific to Europeans and South Asians^15,21^, risk variants in both *ABO* and *FOXP4-AS1* loci are common in worldwide populations^23^. The rare risk variant in *MEF2B*, on the other hand, is specific to East Asians and confers about the eightfold increase in the risk of severe COVID-19 among carriers. These findings, together with many more to discover through ongoing international collaborations^16^, have important implications on the biology underlying the clinical development of COVID-19, and thus might help develop targeted prevention and therapeutic strategies to combat the pandemic.

## Methods

### Ethics statement

This study was reviewed and approved by the Institutional Review Boards of Tongji Hospital (TJ-IRB20200405) and Union Hospital (UH-IRB20200075-1), Tongji Medical College, Huazhong University of Science and Technology, and the Third People’s Hospital of Shenzhen (SZ3H-2020-006-02). Informed consent was obtained from all enrolled patients. Blood samples were collected using the rest of the standard diagnostic tests, with no burden to the patients.

### Phenotype definition

We classified COVID-19 patients into two groups: severe and mild. The severe group included those diagnosed as critical or severe following the guidelines for diagnosis and treatment of COVID-19 (Trial Version 7) released by the National Health Commission of the People’s Republic of China. Briefly, a patient was diagnosed as a critical illness if at least one of the following conditions was met: (1) acute respiratory distress syndrome (ARDS) requiring mechanical ventilation, (2) shock, (3) combining with other organ failure requiring ICU admission. Severe illness was defined by meeting at least one of the following conditions: (1) respiratory rate ≥30 times/min, (2) oxygen saturation ≤93% at resting state, (3) arterial partial pressure of oxygen (PaO_2_)/fraction of inspired oxygen (FiO_2_) ≤300 mmHg, (4) pulmonary imaging examination showed that the lesions significantly progressed by more than 50% within 24–48 h. The other patients, including those with no clinical symptoms, were defined as mild cases. For patients whose electronic medical records (EMR) were available, we examined the EMR to classify the disease severity. For those with no EMR, we extracted information on disease severity from the municipal Notifiable Disease Report System, which might be determined based on similar criteria in early versions of the guidelines for diagnosis and treatment of COVID-19.

### GWAS data of COVID-19

Blood samples of 1626 COVID-19 patients were collected from Tongji Hospital and Hubei Hospital of Traditional Chinese Medicine (TCM) in Wuhan. For samples from Tongji Hospital, genomic DNA was extracted from thawed whole blood samples with the BioTeke Genomic DNA kit (BioTeke, Beijing, China). For samples from Hubei Hospital of TCM, genomic DNA was extracted from 200 µl of EDTA-treated whole blood using the KingFisher Flex Purification System with the KingFisher Pure DNA Blood Kit (Thermo Fisher Scientific). All extractions were performed under Level III protection in the biosafety III laboratories.

For each sample, 200 ng of DNA was loaded on the Infinium Global Screening Array (GSA, Illumina, San Diego, CA) for genotyping, following the manufacturer’s instructions. Genotype calling was performed using GenomeStudio (v 2.0). After filtering SNPs with a call rate < 0.95, we genotyped 650,630 autosomal SNPs and 27,495 SNPs on the X chromosome. We then performed QC using PLINK (v 2.0)^58^, removing 29 samples with missing rate > 0.1, 3 samples with inbreeding coefficient < −0.1, 76 duplicated samples, and 22 samples with the discrepancy between the inferred sex and the recorded sex (Supplementary Fig. 4). Finally, we kept SNPs with Hardy-Weinberg equilibrium (HWE) *P* > 10^−6^, including 649,431 autosomal SNPs and 27,479 X chromosomal SNPs. HWE tests for the X chromosomal SNPs were based on females.

### GWAS data of population controls

We used existing genotyping data of a Coke Oven Worker (COW) cohort in Wuhan as population controls, whose ancestry background is similar to our COVID-19 patient samples collected in Wuhan^59^. In total, 1477 individuals were genotyped using the GSA array in 2018. After excluding 18 samples with call rate < 0.9, 9 potentially contaminated samples (inbreeding coefficient < −0.1), 49 second-degree and above related samples (kinship coefficient φ > 0.088), 1401 individuals (1207 males and 194 females) were included as population controls. We removed SNPs with call rate <0.95, HWE *P* < 10^−6^, and monomorphic variants, and lift over the remaining SNPs from human reference genome GRCh37 to GRCh38, leaving 476,578 autosomal SNPs and 11,082 SNPs on chromosome X.

### Imputation

We merged our COVID-19 GSA dataset and the COW dataset by extracting 436,444 autosomal and 10,528 chromosome X SNPs genotyped in both datasets. We further excluded SNPs with MAF < 0.005 in either dataset, leaving 369,072 autosomal and 8942 chromosome X SNPs for imputation.

We constructed an imputation reference panel by combing the 1000 Genomes Project (1KGP) dataset^23^ and our in-house WGS data of COVID-19 patients from Tongji Hospital (see the “WGS data and analysis” section below). We first extracted the intersecting variants of 1KGP and our WGS dataset and used EAGLE2 (v 2.3.5) to phase our WGS samples with haplotypes from 1KGP as the reference^60^. We then combined the phased haplotypes of our WGS samples and the East Asian samples from 1KGP to form a reference panel to phase and impute our array genotyping datasets. Imputation was performed using Minimac4^61^. For the X chromosome, pseudo-autosomal regions (PARs) were excluded and non-PARs were imputed separately for males and females. After removing variants with MAF < 0.01 and imputation *R*^2^ < 0.8, 6,019,210 autosomal variants and 132,535 on the X chromosome remained for downstream analyses.

### Cryptic relatedness and population structure

We filtered variants with MAF < 0.05, and pruned the merged COVID-19 and COW genotyping data to have linkage disequilibrium (LD) *r*^2^ < 0.5, resulting in 157,968 autosomal SNPs^58^. Using this set of SNPs, we inferred genetic relatedness using KING (v 2.2.5) and determined relatedness types based on the estimated kinship coefficients φ and the probability of zero-IBD-sharing π_0_^62^. We identified 31 first-degree and 7 second-degree related pairs in the COVID-19 samples (Supplementary Fig. 5). After excluding close relatedness up to the second degree (φ > 0.088), we performed principal components analysis (PCA) on the combined COVID-19 and COW datasets. No systematic ancestral or batch effect differences were observed in the top PCs between cases and controls from different sources (Supplementary Fig. 6).

### Association tests and meta-analysis

We performed association analysis using the EPACTS software on the imputed dosage data, which accounts for the imputation uncertainty. After removing close relatedness and 5 patients with missing severity information, we tested for the single-variant association on 598 severe/critical COVID-19 cases versus 2260 controls. Effect sizes and *P* values were derived from Wald tests under a logistic model, adjusting for the first two PCs of population structure. For the analysis of chromosome X, we treated the non-PAR variants as homozygotes for males and included sex as an additional covariate. We also performed genome-wide association analysis further adjusting for age and sex.

We downloaded summary statistics of the COVID-19 HGI B2_release3 analysis of 3199 hospitalized COVID-19 patients versus 897,488 population controls, who were primarily Europeans. There were very few Asian samples in the B2_release3 (only 62 South Asian cases) and we thus did not request for Asian-specific statistics. We performed random-effects meta-analysis using the RE2 model implemented in METASOFT^63^. We chose the B2 dataset rather than the A2 dataset from HGI because the cases in A2 were defined as very severe confirmed COVID-19 cases that required respiratory support more than simple supplementary oxygen, a much stronger case definition than the definition of severe cases in China. Considering that only patients with severe clinical symptoms were recommended for hospitalization in most western countries during the early phase of the pandemic, the case definition of hospitalized COVID-19 patients in B2 dataset aligned better with the severe patient definition in China. We reported meta-analysis results, as well as the *I*^2^ index of heterogeneity across datasets and the corresponding *P*-value *P*_het_. We visualized regional association results using the LocusZoom software with LD information based on Chinese WGS samples^64^.

### WGS data and analysis

Blood samples of 474 severe and 667 mild COVID-19 patients were sequenced at BGI, Shenzhen. These samples were from three sources, including 305 (mild/severe: 211/94) samples from Tongji Hospital in Wuhan, 467 (170/297) from Union Hospital in Wuhan, and 369 (286/83) from the Third People’s Hospital of Shenzhen after excluding related and duplicated samples^22^. For those from Tongji Hospital and the Third People’s Hospital of Shenzhen, genomic DNA was extracted from frozen blood samples using Magnetic Beads Blood Genomic DNA Extraction Kit (MGI, Shenzhen, China). Around 0.5 μg DNA was used for creating the WGS library for each patient. For those from Union Hospital, circulating cell-free DNA (cfDNA) was extracted from 200 μL plasma using MagPure Circulating DNA Mini KF Kit (MD5432-02) following the manufacturer’s instructions. The cfDNA was eluted by 200 μL TE buffer for QC and 40 μL for the rest. The extracted cfDNA was processed to library construction using MGIEasy Cell-free DNA Library Prep kit (MGI, cat. No.: AA00226). After library preparation, all samples were sequenced by the DNBSEQ platform (MGI, Shenzhen, China) to generate 100bp paired-end reads. The mean sequencing depth was 45.0× for those from the Third People’s Hospital of Shenzhen, 33.3× for those from Tongji Hospital, 17.8× for those from Union Hospital.

To minimize batch effects, we performed joint calling and quality controls for all three datasets together. We used Sentieon (sentieon-genomics-201911) for alignment and variant detection following the best practices (https://gatk.broadinstitute.org/hc/en-us/sections/360007226651-Best-Practices-Workflows)^65^. Briefly, sequence reads were mapped to GRCh38 using BWA^66^. For each sample, after duplication removal, INDEL realignment and base quality score recalibration, SNPs and INDELs were detected using the Sentieon Haplotyper algorithm with option “--emit_mode gvcf” to generate an individual GVCF file. Then the GVCF files for all samples were subjected to Sentieon GVCFtyper algorithm for joint variant calling. Variant Quality Score Recalibration (VQSR) was performed using GATK (v 4.1.2). Reference-free LD-based genotype refinement was performed using BEAGLE (v 4.0)^67^, which took the genotype uncertainty into account with the -gl flag. Variants with DR2 < 0.8 or HWE *P* < 10^-6^ were excluded from downstream analysis.

To boost statistical power, we searched for additional population controls from an existing WGS dataset in BGI, Shenzhen, consisting of 1872 unrelated individuals sequenced to a mean depth of 40.0×. Sequencing and genotype calling for this dataset follow the standard WGS protocol as described above except that no LD-based refinement was performed given the high sequencing depth. We combined two call sets by extracting 8,673,249 shared biallelic variants after excluding variants with minor allele counts MAC < 5 or missing rate > 0.05 in either set, or HWE *P* < 10^−6^ in the combined set. We then performed PCA using 539,603 autosomal biallelic SNPs with MAF > 0.05 and LD *r*^2^ < 0.5^58^. For each of our 474 severe COVID-19 cases, we identified two ancestry-matched population controls based on Euclidean distances in the first two PCs using the *optmatch* R package^68,69^. Thus, the final WGS dataset consists of 474 cases with severe symptoms, 667 controls with mild symptoms, and 948 ancestry-matched populations controls. Again, no systematic differences between samples from different batches were found in the top PCs (Supplementary Fig. 7).

Despite the very stringent quality controls described above, residual batch effects might persist because samples from Union Hospital were sequenced at 17.8× using cfDNA. We, therefore, included an indicator variable for the cfDNA batch and the first two PCs as covariates in our logistic model for association tests between 474 severe cases and 1615 controls (mild patients and population controls). Genomic inflation factor was *λ*_GC_ = 1.001, indicating overall well-controlled batch effects and population stratification. Furthermore, we performed additional association tests on 667 mild patients versus 948 population controls, adjusting for the top two PCs and the cfDNA batch indicator, and identified 1869 variants with *P* < 10^−6^. We conservatively excluded these variants from our final results because they might be subject to residual batch effects.

### Clinical measurements

We analyzed the serum level of inflammatory biomarkers, including interleukin 1 beta (IL-1β), interleukin 2 receptor (IL-2R), interleukin 6 (IL-6), interleukin 8 (IL-8), interleukin 10 (IL-10), tumor necrosis factor-alpha (TNF-α), complements C3 and C4, and C-reactive protein (CRP) for hospitalized patients from Tongji Hospital, including both mild and severe patients. For patients with measurements at multiple time points, we used the earliest measurement after hospitalization. The majority of the measurements were taken in the first week of hospitalization. We compared these clinical measurements in different groups of samples defined by COVID-19 severity, age, sex, and genotypes of GWAS top SNPs using the Wilcoxon rank-sum test and Spearman’s correlation.

### Reporting summary

Further information on research design is available in the Nature Research Reporting Summary linked to this article.

## Supplementary information

Supplementary Information

Description of Additional Supplementary File

Supplementary Data 1

Reporting Summary

## Data Availability

Summary statistics of the association tests in our Chinese samples have been deposited in the China National Genebank Sequence Archive (https://db.cngb.org/cnsa/) with accession number CNP0001981. Individual-level genotype data are not publicly available due to the protection of privacy and regulations. Source data for Fig. 2 is provided in Supplementary Data 1.

## Code availability

Details regarding the packages and versions used are included in “Methods”.
