# Supplementary material for: Trans-ethnic genome-wide association study of severe COVID-19
Source: Commun Biol. 2021 Aug 31;4:1034. doi: 10.1038/s42003-021-02549-5 (PMC8408224; doi:10.1038/s42003-021-02549-5)
Supplement: Supplementary file 1 — Supplementary Information [file 42003_2021_2549_MOESM1_ESM.pdf]

Supplementary Materials for

Trans-ethnic genome-wide association study of severe COVID-19

Peng Wu[1,2,*], Lin Ding[3,4,*], Xiaodong Li[5,6,*], Siyang Liu[7,*], Fanjun Cheng[8,*], Qing He[9,*], Mingzhong Xiao[5,6], Ping Wu[1,2], Hongyan Hou[2,10], Minghui Jiang[3,4], Pinpin Long[4,11], Hao Wang[4,11], Linlin Liu[12], Minghan Qu[3,4], Xian Shi[3,4], Qin Jiang[4,11], Tingting Mo[4,11], Wencheng Ding[1,2], Yu Fu[1,2], Shi Han[12], Xixiang Huo[12], Yingchun Zeng[12], Yana Zhou[5,6], Qing Zhang[5,6], Jia Ke[5,6], Xi Xu[5,6], Wei Ni[5,6], Zuoyu Shao[5,6], Jingzhi Wang[5,6], Panhong Liu[13], Zilong Li[13], Yan Jin[14], Fang Zheng[15], Fang Wang[9], Lei Liu[9], Wending Li[4,11], Kang Liu[4,11], Rong Peng[4,11], Xuedan Xu[4,11], Yuhui Lin[4,11], Hui Gao[4,11], Limei Shi[4,11], Ziyue Geng[4,11], Xuanwen Mu[4,11], Yu Yan[3,4], Kai Wang[3,4], Degang Wu[3,4], Xingjie Hao[3,4], Shanshan Cheng[3,4], Gaokun Qiu[4,11], Huan Guo[4,11], Kezhen Li[1,2], Gang Chen[1,2], Ziyong Sun[2,10], Xihong Lin[16,17,18], Xin Jin[19,#], Feng Wang[2,10,#], Chaoyang Sun[1,2,#], Chaolong Wang[2,3,4,#]

Supplementary Table 1. Comparison of top association loci with and without adjustment for age and sex in the analyses of Chinese samples.

Lead variant	Dataset	No adjustment for age and sex			Adjusting for age and sex		
		**OR (95% CI)†**	***P***	**Heterogeneity**	**OR (95% CI)†**	***P***	**Heterogeneity**
rs1853837 at 6p21.1	Chinese (GWAS)	1.30 (1.13-1.50)	3.24×10^{-4}		1.33 (1.12-1.57)	9.48×10^{-4}	
	HGI (B2_release3)	1.28 (1.15-1.42)	5.24×10^{-6}		1.28 (1.15-1.42)	5.24×10^{-6}	
	Chinese (WGS)	1.27 (1.07-1.51)	7.06×10^{-3}	I^2=0.00%	1.30 (1.08-1.57)	6.24×10^{-3}	I^2=0.00%
	Meta-analysis	1.28 (1.19-1.39)	2.51×10^{-10}	P_{het}=0.97	1.29 (1.19-1.40)	4.20×10^{-10}	P_{het}=0.93
rs8176719 at 9q34.2	Chinese (GWAS)	1.28 (1.12-1.46)	3.19×10^{-4}		1.15 (0.99-1.35)	6.80×10^{-2}	
	HGI (B2_release3)	1.17 (1.09-1.26)	1.27×10^{-5}		1.17 (1.09-1.26)	1.27×10^{-5}	
	Chinese (WGS)	1.17 (0.98-1.38)	8.03×10^{-2}	I^2=0.00%	1.17 (0.97-1.41)	1.08×10^{-1}	I^2=0.00%
	Meta-analysis	1.19 (1.12-1.26)	8.98×10^{-9}	P_{het}=0.51	1.17 (1.10-1.24)	5.88×10^{-7}	P_{het}=0.99
rs74490654 at 19q13.11	Chinese (WGS)	8.73 (4.14-18.41)	1.22×10^{-8}	-	10.93 (4.56-26.19)	8.22×10^{-8}	-

Notes: †Odds ratio (OR) and 95% confidence interval (CI) of the alternative allele. Meta-analysis is based on the Han-Eskin random-effect method. All association analyses of Chinese samples have adjusted for the top two PCs.

Supplementary Table 2. Suggestive loci associated with COVID-19 severity ($P<10^{-6}$).

Locus	Dataset	Sample size	Lead variant	AF$	OR (95% CI) †	*P*	Heterogeneity
21q22.11	Chinese (GWAS)	598/2,260	rs1051393	0.610	1.12 (0.97-1.28)	1.16×10^{-1}	
IFNAR2	HGI (B2_release3)	3,199/897,488	chr21: 33241950	0.333	1.19 (1.11-1.27)	1.12×10^{-6}	
	Chinese (WGS)	474/1,615	T/G	-	-	-	I^2=0.00%
	Meta-analysis	3,797/899,748	Missense		1.17 (1.10-1.25)	4.33×10^{-7}	P_{het}=0.43
3p14.2	Chinese (GWAS)	598/2,260	rs672699	0.478	1.04 (0.91-1.19)	5.80×10^{-1}	
PTPRG	HGI (B2_release3)	3,199/897,488	chr3:61768231	0.789	1.19 (1.10-1.29)	1.36×10^{-5}	
	Chinese (WGS)	474/1,615	T/A	0.484	1.37 (1.14-1.63)	5.49×10^{-4}	I^2=67.21%
	Meta-analysis	4,271/901,363	Intronic		1.18 (1.04-1.34)	5.58×10^{-7}	P_{het}=0.05
16q21	Chinese (GWAS)	598/2,260	rs7499679	0.250	0.85 (0.72-0.99)	3.80×10^{-2}	
ADGRG1	HGI (B2_release3)	3,199/897,488	chr16:57636629	0.227	0.86 (0.79-0.92)	5.92×10^{-5}	
	Chinese (WGS)	474/1,615	G/A	0.276	0.80 (0.66-0.97)	2.65×10^{-2}	I^2=0.00%
	Meta-analysis	4,271/901,363	Intronic		0.85 (0.79-0.90)	8.09×10^{-7}	P_{het}=0.82
1q44	Chinese (GWAS)	598/2,260	rs12130553	-	-	-	
HNRNPU	HGI (B2_release3)	3,199/897,488	chr1:244873270	0.437	1.19 (1.11-1.28)	4.19×10^{-6}	
	Chinese (WGS)	474/1,615	T/C	0.338	1.18 (0.99-1.41)	6.91×10^{-2}	I^2=0.00%
	Meta-analysis	3,673/899,103	Intergenic		1.19 (1.11-1.27)	9.17×10^{-7}	P_{het}=0.93

Notes: This table presents loci that are included in at least two datasets and have meta-analysis *P* value between 5×10^{-8} and 10^{-6}. Sample size is presented as number of cases / number of controls. * Variant with the smallest p value within each locus: rs number, GRCh38 genomic position, reference/alternative alleles, annotation. $ AF: frequency of the alternative allele: from top to down is the AF in Chinese GWAS controls, the AF in 1KGP European samples, and the AF in Chinese WGS controls. †Odds ratio (OR) and 95% confidence interval (CI) of the alternative allele. Meta-analysis is based on random-effect model.

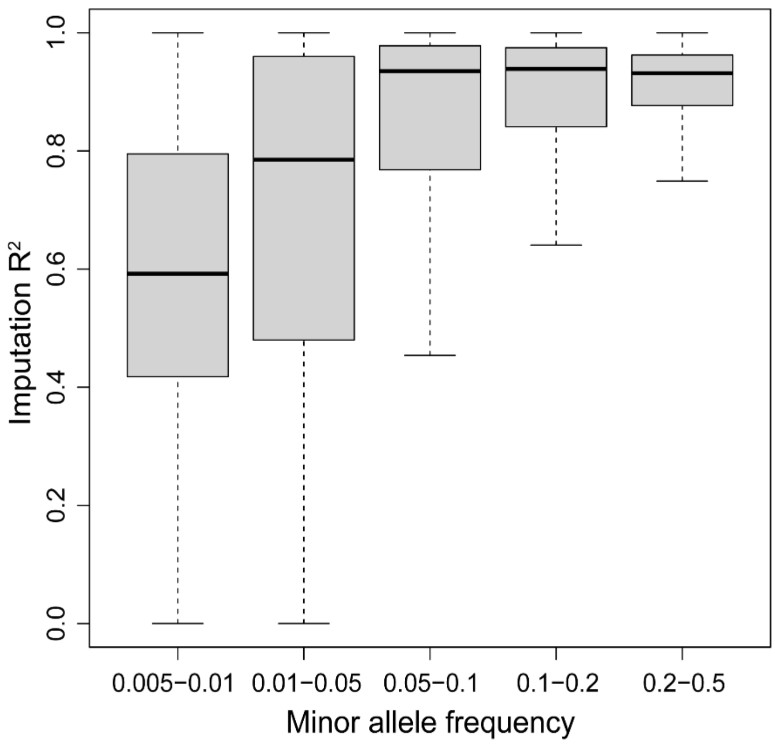

Supplementary Figure 1. Imputation quality as a function of MAF. Each box summarizes the imputation R^2 for autosomal SNPs within a MAF bin. The dark horizontal line represents median value, and the grey box represents interquartile range (IQR). Outliers below the lower whiskers (1.5×IQR below the 25th percentile) of the last three bins are not shown.

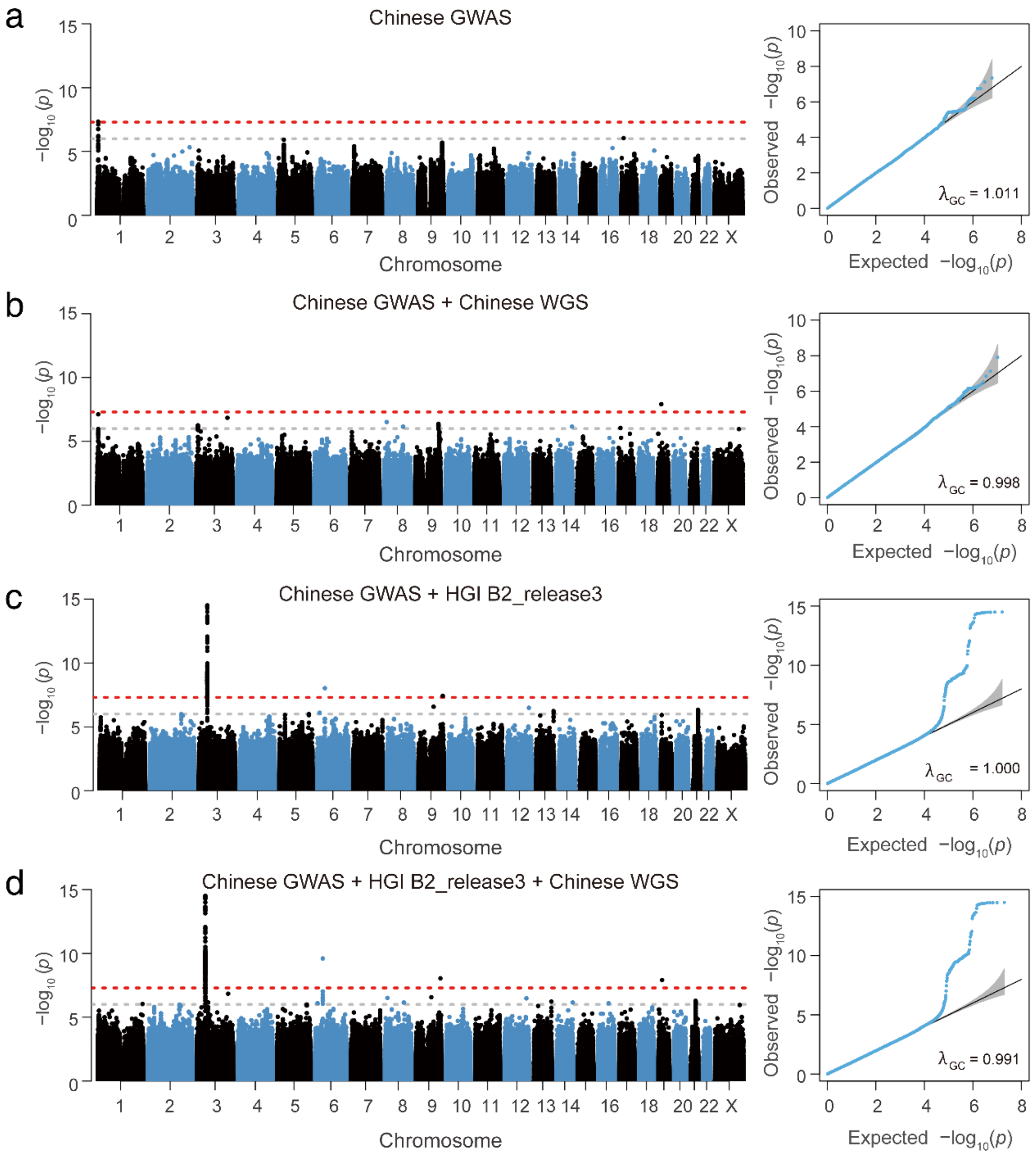

Supplementary Figure 2. GWAS results for severe COVID-19 with first two PCs included as covariates. (a) Manhattan and QQ plots for Chinese GWAS. (b) Manhattan and QQ plots for the meta-analysis of Chinese GWAS and Chinese WGS results. (c) Manhattan and QQ plots for the meta-analysis of Chinese GWAS and HGI B2_release3 results. (d) Manhattan and QQ plots for the meta-analysis of Chinese GWAS, HGI B2_release3 results, and Chinese WGS. In Manhattan plots, the red dash line indicates genome-wide significance level of P=5×10^{-8} and the grey dash line indicates suggestive significance level of P=10^{-6}. In QQ plots, the grey region represents the 95% CI of P values under the null hypothesis of no association.

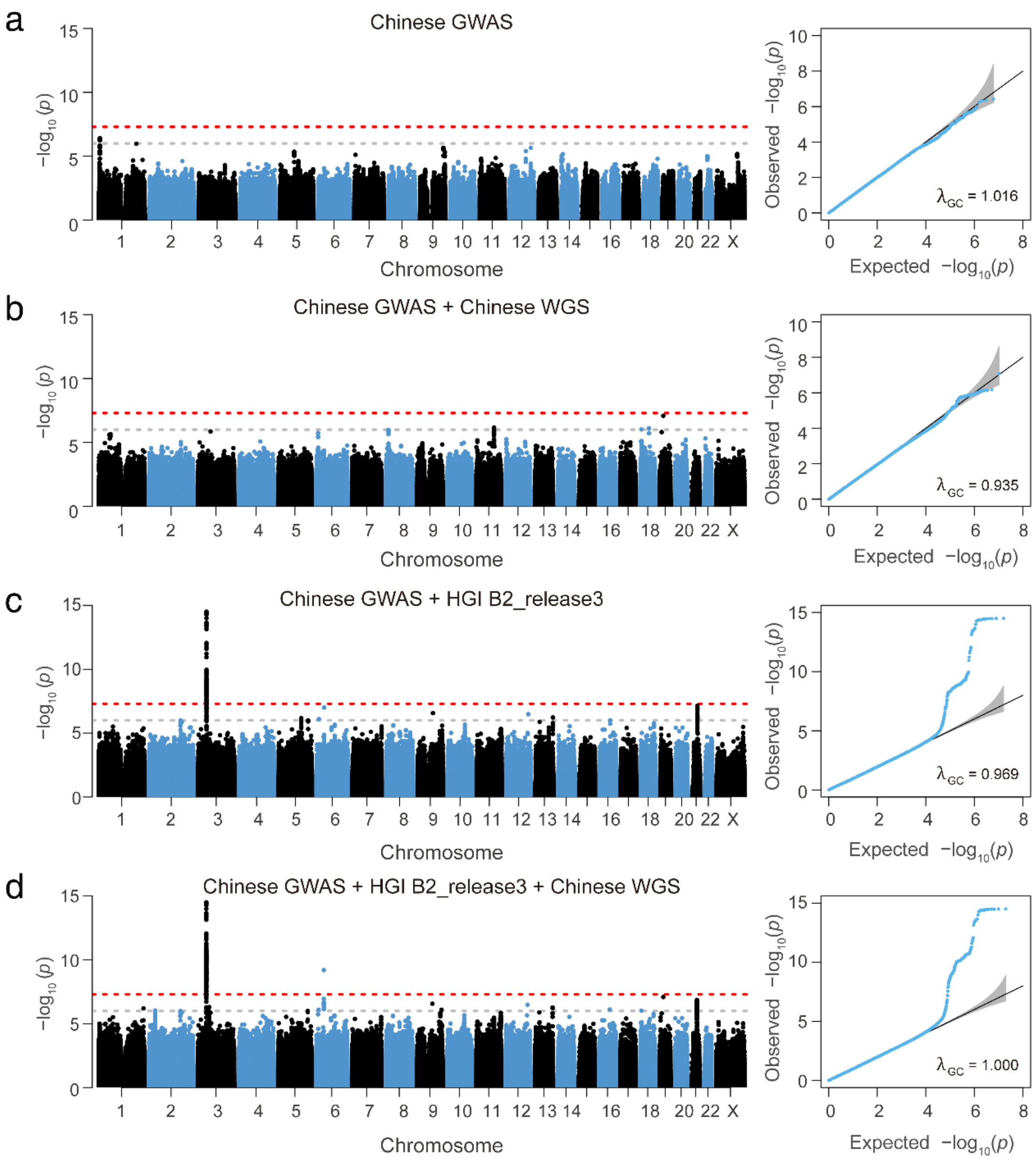

Supplementary Figure 3. GWAS results for severe COVID-19 with first two PCs, age and sex included as covariates. (a) Manhattan and QQ plots for Chinese GWAS. (b) Manhattan and QQ plots for the meta-analysis of Chinese GWAS and Chinese WGS results. (c) Manhattan and QQ plots for the meta-analysis of Chinese GWAS and HGI B2_release3 results. (d) Manhattan and QQ plots for the meta-analysis of Chinese GWAS, HGI B2_release3 results, and Chinese WGS. In Manhattan plots, the red dash line indicates genome-wide significance level of P=5×10^{-8} and the grey dash line indicates suggestive significance level of P=10^{-6}. In QQ plots, the grey region represents the 95% CI of P values under the null hypothesis of no association.

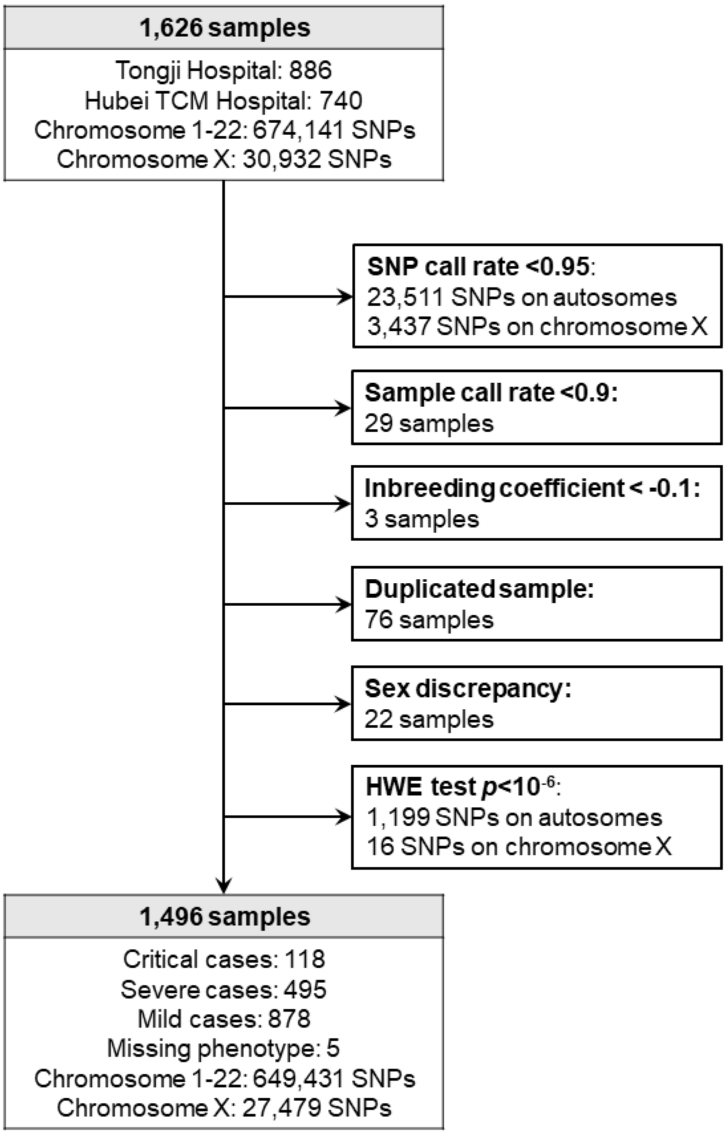

Supplementary Figure 4. Quality control of the GWAS array data of COVID-19 patients. Hubei TCM Hospital: Hubei Hospital of Traditional Chinese Medicine.

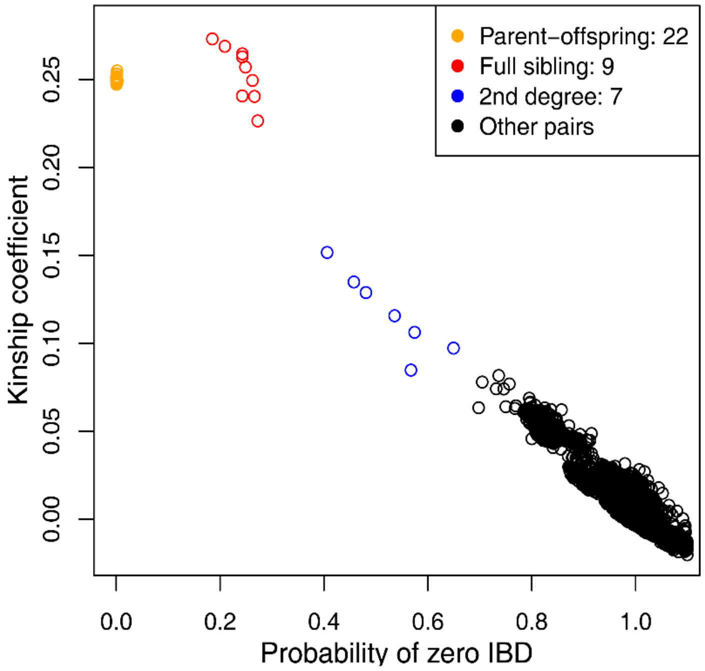

Supplementary Figure 5. Cryptic relatedness in the GSA genotyped COVID-19 samples. Numbers of pairs for each relatedness type were presented in the legend. The x-axis is the probability of sharing 0 alleles identical-by-descent (IBD) at a SNP between two individuals.

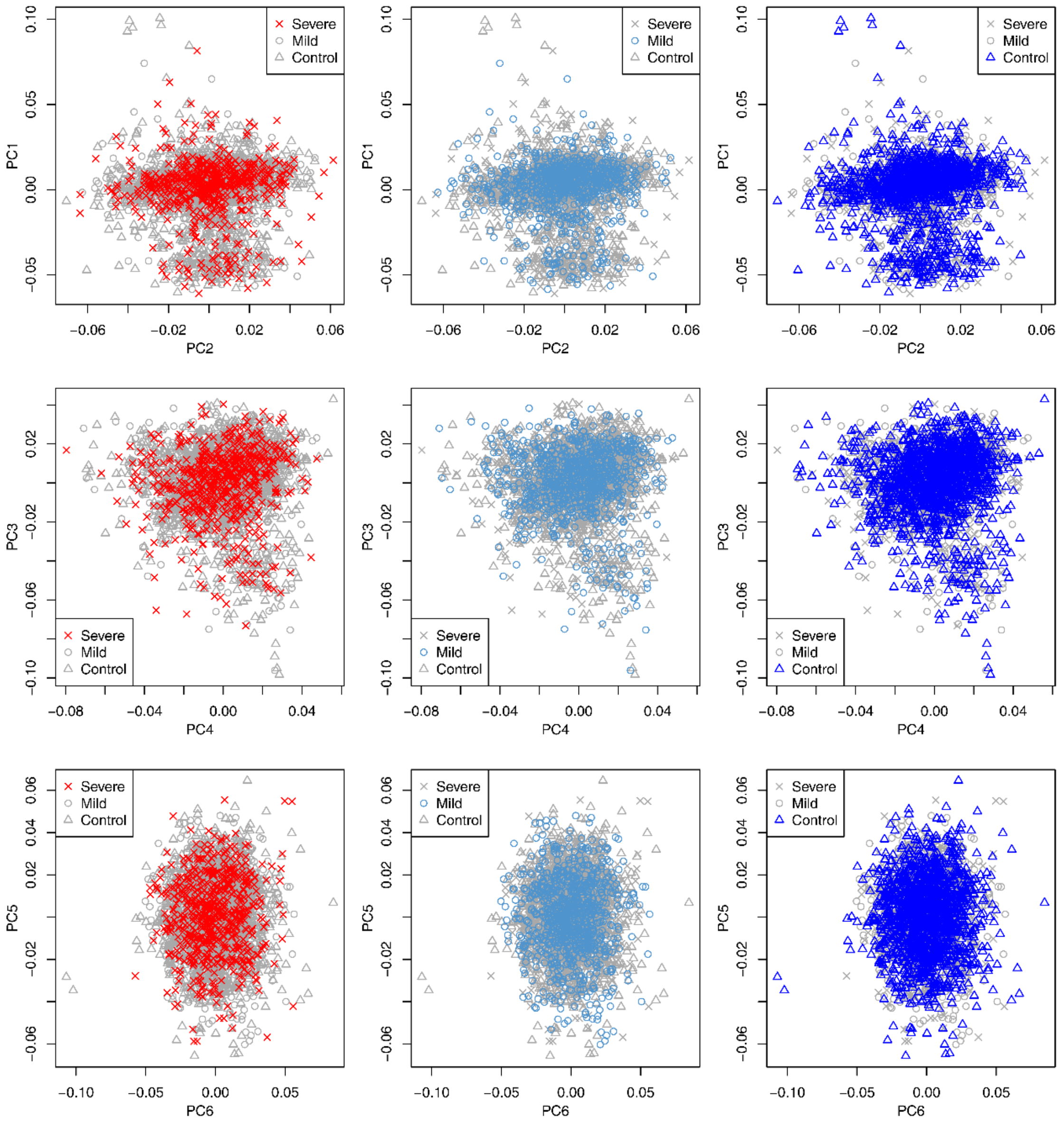

Supplementary Figure 6. PCA of the Chinese GWAS samples. We highlighted samples severe COVID-19, mild COVID-19, and ancestry-matched population controls in columns from left to right, respectively.

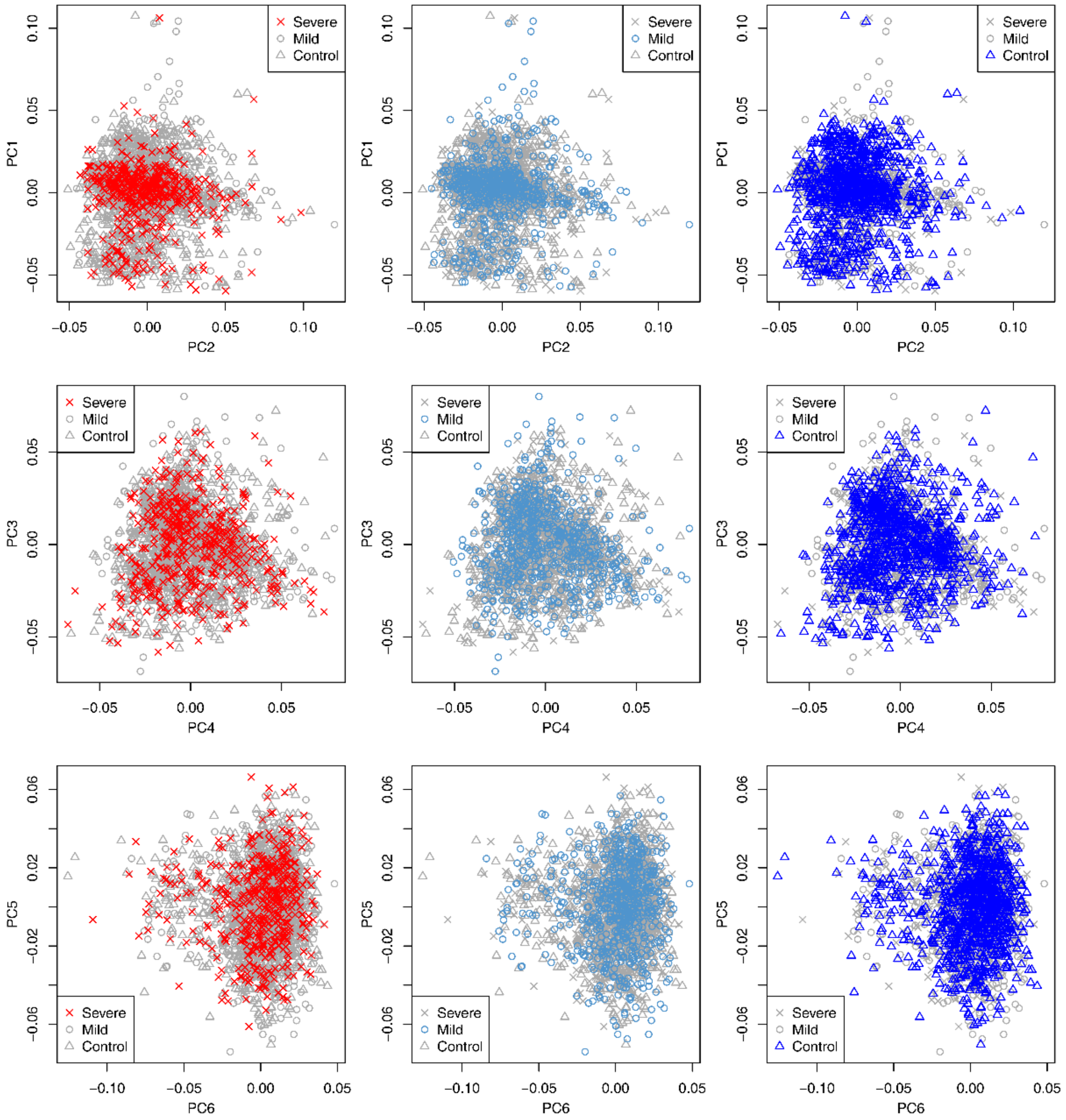

Supplementary Figure 7. PCA of Chinese WGS samples. We highlighted samples severe COVID-19, mild COVID-19, and ancestry-matched population controls in columns from left to right, respectively.
