# Supplementary material for: Trans-ethnic genome-wide association study of severe COVID-19
Source: Commun Biol. 2021 Aug 31;4:1034. doi: 10.1038/s42003-021-02549-5 (PMC8408224; doi:10.1038/s42003-021-02549-5)
Supplement: Supplementary file 2 — Description of Additional Supplementary File [file 42003_2021_2549_MOESM2_ESM.pdf]

Description of additional supplementary file

Supplementary Data 1: Source data for Figure 2, “Comparison of serum level of IL-1β in different groups”.
