# Supplementary material for: Trans-ethnic genome-wide association study of severe COVID-19
Source: Commun Biol. 2021 Aug 31;4:1034. doi: 10.1038/s42003-021-02549-5 (PMC8408224; doi:10.1038/s42003-021-02549-5)
Supplement: Supplementary file 4 — Reporting Summary [file 42003_2021_2549_MOESM4_ESM.pdf]

nature research

Corresponding author(s): Xin Jin, Feng Wang, Chaoyang Sun, Chaolong Wang

Last updated by author(s): Jul 29, 2021

Reporting Summary

Nature Research wishes to improve the reproducibility of the work that we publish. This form provides structure for consistency and transparency in reporting. For further information on Nature Research policies, see our Editorial Policies and the Editorial Policy Checklist.

Statistics

For all statistical analyses, confirm that the following items are present in the figure legend, table legend, main text, or Methods section.

n/a	Confirmed	
☐	☒	The exact sample size (*n*) for each experimental group/condition, given as a discrete number and unit of measurement
☒	☐	A statement on whether measurements were taken from distinct samples or whether the same sample was measured repeatedly
☐	☒	The statistical test(s) used AND whether they are one- or two-sided<br>*Only common tests should be described solely by name; describe more complex techniques in the Methods section.*
☐	☒	A description of all covariates tested
☐	☒	A description of any assumptions or corrections, such as tests of normality and adjustment for multiple comparisons
☐	☒	A full description of the statistical parameters including central tendency (e.g. means) or other basic estimates (e.g. regression coefficient) AND variation (e.g. standard deviation) or associated estimates of uncertainty (e.g. confidence intervals)
☐	☒	For null hypothesis testing, the test statistic (e.g. F, t, r) with confidence intervals, effect sizes, degrees of freedom and P value noted<br>*Give P values as exact values whenever suitable.*
☒	☐	For Bayesian analysis, information on the choice of priors and Markov chain Monte Carlo settings
☒	☐	For hierarchical and complex designs, identification of the appropriate level for tests and full reporting of outcomes
☐	☒	Estimates of effect sizes (e.g. Cohen's *d*, Pearson's *r*), indicating how they were calculated

Our web collection on statistics for biologists contains articles on many of the points above.

Software and code

Policy information about availability of computer code

Data collection	GenomeStudio (v 2.0) was used to perform genotype calling for the GWAS array genotyping data.<br>Sentieon (sentieon-genomics-201911) and GATK (v 4.1.2) were used for alignment and genotype calling of sequencing data.
Data analysis	Phasing and genotype imputation were performed using BEAGLE (v 4.0), EAGLE2 (v 2.3.5) and Minimac4.<br>Quality controls, association tests, and meta-analysis were performed using PLINK (v 2.0), KING (v 2.2.5), EPACTS (v 3.3.0), and METASOFT (v 2.0.1).<br>Graph visualization and simple statistical tests were performed using LocusZoom (v 1.4) and R (v 4.0.0).

For manuscripts utilizing custom algorithms or software that are central to the research but not yet described in published literature, software must be made available to editors and reviewers. We strongly encourage code deposition in a community repository (e.g. GitHub). See the Nature Research guidelines for submitting code & software for further information.

Data

Policy information about availability of data

All manuscripts must include a data availability statement. This statement should provide the following information, where applicable:

- Accession codes, unique identifiers, or web links for publicly available datasets
- A list of figures that have associated raw data
- A description of any restrictions on data availability

Summary statistics of the association tests in our Chinese samples have been deposited in the China National Genebank Sequence Archive (https://db.cngb.org/cnsa/) with accession number CNP0001981. Individual-level genotype data are not publicly available due to protection of privacy and regulations.

Field-specific reporting

Please select the one below that is the best fit for your research. If you are not sure, read the appropriate sections before making your selection.

☒ Life sciences ☐ Behavioural & social sciences ☐ Ecological, evolutionary & environmental sciences

For a reference copy of the document with all sections, see nature.com/documents/nr-reporting-summary-flat.pdf

Life sciences study design

All studies must disclose on these points even when the disclosure is negative.

Sample size	We genotyped 1,457 (598/859 with severe/mild symptoms) and sequenced 1,141 (severe/mild: 474/667) patients of Chinese ancestry. We further incorporated 1,401 genotyped and 948 sequenced ancestry-matched population controls, and tested genome-wide association on 1,072 severe cases versus 3,875 mild or population controls, followed by trans-ethnic meta-analysis with summary statistics of 3,199 hospitalized cases and 897,488 population controls from the COVID-19 Host Genetics Initiative.
Data exclusions	We excluded samples that have low data quality (call rate <0.9), potential contamination (inbreeding coefficient <-0.1), duplication or close relatedness up to 2nd degree, sex discrepancy between genotype and phenotype data, and missing phenotypes.<br>SNPs were excluded followed the standard QC procedures based on Hardy-Weinberg equilibrium tests (P<1e-6), call rate (<0.95), low imputation quality (R2<0.8), and minor allele frequency (MAF<0.01).
Replication	Our association analyses were based on trans-ethnic meta-analysis of three different datasets of large sample sizes to ensure reproducibility of the signals across datasets. We were not able to reproduce a Chinese specific rare-variant association signal due to lack of more cases in China and this limitation has been described in the manuscript.
Randomization	Not applicable
Blinding	Not applicable

Reporting for specific materials, systems and methods

We require information from authors about some types of materials, experimental systems and methods used in many studies. Here, indicate whether each material, system or method listed is relevant to your study. If you are not sure if a list item applies to your research, read the appropriate section before selecting a response.

Materials & experimental systems

n/a	Involved in the study	
☒	☐	Antibodies
☒	☐	Eukaryotic cell lines
☒	☐	Palaeontology and archaeology
☒	☐	Animals and other organisms
☐	☒	Human research participants
☒	☐	Clinical data
☒	☐	Dual use research of concern

Methods

n/a	Involved in the study	
☒	☐	ChIP-seq
☒	☐	Flow cytometry
☒	☐	MRI-based neuroimaging

Human research participants

Policy information about studies involving human research participants

Population characteristics	Population characteristics of the samples included in our analyses were presented in Table 1 of the paper.
Recruitment	The Chinese COVID-19 patients were laboratory-confirmed and hospitalized patients. We included all participants with informed consent and available blood sample remains from routine blood tests. Population controls were from existing GWAS and sequencing datasets and were ancestry-matched to the cases to avoid potential bias due to population structure.
Ethics oversight	This study was reviewed and approved by the Institutional Review Boards of Tongji Hospital (TJ-IRB20200405) and Union Hospital (UH-IRB20200075-1), Tongji Medical College, Huazhong University of Science and Technology, and the Third People's Hospital of Shenzhen (SZ3H-2020-006-02).

Note that full information on the approval of the study protocol must also be provided in the manuscript.
